# Context-Unsupervised Adversarial Network for Video Sensors †

**DOI:** 10.3390/s22093171

**Published:** 2022-04-21

**Authors:** Gemma Canet Tarrés, Montse Pardàs

**Affiliations:** Image Processing Group, Department of Signal Theory and Communications, Universitat Politècnica de Catalunya (UPC), 08034 Barcelona, Spain; g.canettarres@surrey.ac.uk

**Keywords:** background subtraction, adversarial networks, deep learning, computer vision, video sensors

## Abstract

Foreground object segmentation is a crucial first step for surveillance systems based on networks of video sensors. This problem in the context of dynamic scenes has been widely explored in the last two decades, but it still has open research questions due to challenges such as strong shadows, background clutter and illumination changes. After years of solid work based on statistical background pixel modeling, most current proposals use convolutional neural networks (CNNs) either to model the background or to make the foreground/background decision. Although these new techniques achieve outstanding results, they usually require specific training for each scene, which is unfeasible if we aim at designing software for embedded video systems and smart cameras. Our approach to the problem does not require specific context or scene training, and thus no manual labeling. We propose a network for a refinement step on top of conventional state-of-the-art background subtraction systems. By using a statistical technique to produce a rough mask, we do not need to train the network for each scene. The proposed method can take advantage of the specificity of the classic techniques, while obtaining the highly accurate segmentation that a deep learning system provides. We also show the advantage of using an adversarial network to improve the generalization ability of the network and produce more consistent results than an equivalent non-adversarial network. The results provided were obtained by training the network on a common database, without fine-tuning for specific scenes. Experiments on the unseen part of the CDNet database provided 0.82 a F-score, and 0.87 was achieved for LASIESTA databases, which is a database unrelated to the training one. On this last database, the results outperformed by 8.75% those available in the official table. The results achieved for CDNet are well above those of the methods not based on CNNs, and according to the literature, among the best for the context-unsupervised CNNs systems.

## 1. Introduction

Surveillance cameras are nowadays ubiquitous in multiple scenarios. It is estimated than over one billion cameras will be in use in the next few years, according to a report from IHS Markit [1], both for outdoor and indoor surveillance. Thus, there is much interest in the development of computer vision software to automatically process the videos captured and extract high level information from these video sensors. While computer vision techniques are currently monopolized by deep learning, a further step needs to be done to make them usable in a very wide variety of scenarios, such us those encountered in surveillance.

We focus on a first step of video analysis, which is the process of identifying each pixel in every image as part of foreground or background, usually known as “foreground segmentation”. It is also referred as “moving object detection”, since we usually identify foreground objects as those scene objects which do not follow the camera’s motion. Foreground segmentation is widely employed for many applications, such as detecting intruding objects, automatically monitoring large and crowded areas (such as airports), and traffic monitoring of roads. It is also a very useful tool as a first processing step that provides focus of attention for tracking, recognition, analyzing human activities, or editing videos by changing or isolating their backgrounds.

This problem has been widely studied during the last two decades, with many different contributions. Classical methods such as [2,3,4,5,6,7,8,9,10,11,12,13,14] are based on modeling the background per-pixel distribution and identifying the foreground pixels that are exceptions in their corresponding background models. Recently, deep neural networks have been used both for modeling the background and for the subtraction step [15,16,17,18,19,20]. Among them, a few explore the usage of adversarial networks. While outstanding results are obtained using neural networks, these methods use a training or fine-tuning step based on the sequence to evaluate. In this paper, the possibilities of using neural networks for background subtraction without the need for training or fine-tuning on the testing scenes are explored, as in real scenarios it is not feasible to require user-provided detailed segmentation for a large number of frames, or even to devote a few hours to training the network with unlabeled background. The aim is to build a system that can be used in very different scenarios without adaptation.

To approach what can be called a foreground segmentation context-unsupervised, meaning that training does not use the testing scenes, we propose to use a two-step scheme. The first step uses a statistical foreground detection system that produces a rough approximation to the foreground. These classic systems have the possibility to accommodate to different requirements of the application scenario, such as strong shadows, multi-modal background, or delay in incorporating moving objects into the background. However, their results in a global framework are always below the ones obtained with supervised methods that use the ground truth for training, as shown in the Results section of www.changedetection.net. Thus, in a second step, the output of this system as input to a refining convolutional neural network is used. It is based on a semantic segmentation network which is trained to accurately segment the objects that are present in an input mask.

In our previous work [21], we proposed a refinement network based on a U-Net semantic segmentation network. However, taking into account that previous supervised approaches to background segmentation have used conditional GANs [20], we explore in this paper the usage of an adversarial network. Adversarial networks have been shown in [22] to produce better results for semantic segmentation, while improving the generalization properties of the network to unseen data. This is particularly important in our problem, where labeled databases are scarce, and there is a high variability between training and testing scenarios. According to [22], the adversarial network can be considered as a “variational” loss, with adjustable parameters, which can regularize the segmentation model by enforcing higher-order consistency between the ground truth segmentation maps and the network output.

Additionally, conditional generative adversarial networks (cGANs) [23] have been introduced as a special case of generative adversarial networks in which an additional input is given to both the generator and discriminator networks. Impressive results have been achieved by cGANS in many image-to-image transformation problems, such as in-painting, style transfer, and domain adaptation. However, its applications to vision tasks where the outputs are less complex than the inputs, such as segmentation, have so far produced worse results than those obtained with simpler networks and a more direct loss function. In this work we explore its use in the context of a semantic segmentation network used as a refinement step.

The main contributions of this work can be summarized as follows:

- An algorithm that can be integrated in video sensors and used in a wide variety of scenarios without requiring context adjustment is proposed.
- The two-step scheme proposed for foreground segmentation provides results superior to the ones obtained by conventional, non-learning based methods, using a convolutional neural network which does not require specific scene training.
- An image-to-image translation network is adapted to be used as a refinement network, by conditioning the output not only on the input image, but also on the rough mask obtained by the statistical based background subtraction.
- It is shown that, in a vision task, it is also advantageous to model the loss function with an adversarial network in order to provide better generalization and accuracy.
- An example of how learning based techniques can be used in conjunction with non-learning methods is provided. This allows one to introduce high level features according to the application and scenario, which currently cannot be used in most deep learning systems.

The remainder of the paper is organized as follows. Section 2 describes the state-of-the-art of foreground segmentation, Section 3 describes the pipeline and the networks used, Section 4 presents the results obtained, and Section 5 and Section 6 discuss the results and conclude the paper.

## 2. Related Works

Before the introduction of our foreground detection system, the related works on foreground detection are reviewed, both with classical techniques and with deep learning. In the second case, we focus on systems using generative adversarial networks.

### 2.1. Classical Approaches to Foreground Detection by Background Modeling and Subtraction

One of the fundamental problems of video segmentation is differentiating foreground objects from their background. A classical approach for achieving this consists of two steps: the modeling of background and the comparison of this model with the current frame for classifying each pixel as foreground or background.

Background is sometimes modeled as a single image computed as the temporal average or the median of a set of frames [2,3,4]. However, most methods do not model it as just a single image, but using a more complex, probabilistic approach, capable of handling challenging situations such as illumination changes or slight variations due to outdoors conditions. Some examples for that are the Gaussian mixture model (GMM) [5] and the kernel density estimation (KDE) [6]. In the first one, each background pixel is modeled as a random variable, affected by noise and scene variability, and whose probability density function (PDF) can be modeled as a mixture of Gaussian functions; in the second, each background pixel’s PDF is estimated by the histogram of the N most recent pixel values, each smoothed by using a Gaussian kernel. Currently, the methods that obtain the best results for background modeling, LaBGen [7] and motion-assisted spatio-temporal clustering of low-rank (MSCL) [8], are based on robust median estimation and robust principal component analysis (PCA), respectively. Joint background modeling and subtraction has been approached in many different ways. One of the first introduced methods, still widely used today, is the one of Stauffer and Grimson [5]. This method models each pixel PDF as a mixture of Gaussians and uses an on-line approximation to update the model. By determining the Gaussian distributions that correspond to the background, each pixel can be labeled as foreground or background.

The great performance of that algorithm gave rise to a series of different methods that employ slight variations to improve its weakest points. Focusing on its slow learning when the initialization is performed in busy environments and its big struggle of identifying shadows of moving objects, Kaewtrakulpong and Bowden [9] proposed a method based on the same principles as [5] but with some adaptations in the updating equations. Additionally, they proposed a postprocessing algorithm based on color distortion comparison for reducing the number of shadows detected. A few years later, Zivkovic [10,11] developed a similar variation from the original Stauffer and Grimson algorithm, based on swapping the equations for recursive ones and supplying the model with an automatic selection for the optimal number of Gaussians in each case.

However, not all classical approaches are parametric nor based on Gaussians. One example for these alternative methods is the one of Godbehere, Matsukawa, and Goldberg [12] that directly estimates the distribution for all background pixels instead of its parameters, thereby performing in a non-parametric way and making use of the pixel’s histogram distribution. Another non-parametric method for moving object detection is that presented by Cuevas and García [13], which achieves high quality detections even in complex background scenarios and for non-completely static scenes, by dynamically estimating the bandwidth of the kernels used in the modeling part and selectively updating the background model. Later on, this method is improved [14] by modeling not only the background, but also the foreground. At each new frame, for this approach, the spatial positions of the foreground data are updated using a particle filter that predicts its most probable movement.

### 2.2. Adversarial Networks

Generative adversarial networks (GANs) [24] were first introduced in 2014 by Goodfellow et al., as a framework to estimate deep generative models via an adversarial process, and they became a big breakthrough in the field of unsupervised learning. They did not only provide a tool for training machine learning models using unlabeled data, but also changed the vision of learning based on a loss function. A GAN consists on two different modules: the first one, the generator, which tries to capture the distribution of data, creating a new image, and a discriminator, which takes this generated image as input, and decides on its authenticity: either it is the expected target or a synthetic approximation. While traditional CNN approaches require a good design of effective loss, with GANs, only the high level goal of the network has to be provided, and the loss function for satisfying it is automatically learned. This loss is a structured one, which can penalize the joint configuration of the output if it differs from the target, rather than considering each pixel independently from the others.

The advantages of using adversarial networks have been shown in [22] for a semantic segmentation task. While in the original work [24] the adversarial model was used to define a loss for a generative model in which the calculation of the cross-entropy loss is intractable, the CNN-based segmentation models used in [22] allow one to compute the exact multi-class cross-entropy loss. In this scenario, the discriminative network is only used to regularize the segmentation model, which as the authors show, helps to improve the results and the generalization properties of the network to unseen data.

A similar scheme arose from the application of conditional GANs (cGANs) [25]. The GANs structure from [24] was extended by adding another input to both generator and discriminator, in order to learn conditional distributions. In this case, the output of the generator is an image that is somehow related to the additional input. In particular, if the extra information is an image, it is a case of image-to-image translation. Presented in 2016, *pix2pix* [23] is a project that aims at showing multiple applications of this transformation, demonstrating the versatility of cGANs for performing multiple tasks. The explored examples include colorizing images to transform sketches into images, converting semantic labels into photos, the inpainting of photos with missing pixels, and the semantic segmentation of images, among other applications. This last task, however, being the only task where the output is less complex than the input, does not show in this work as good results as the others. In this case, reconstruction losses such as L1 were shown to be mostly sufficient.

### 2.3. Deep Learning Approaches to Foreground Detection

Attempts to obtain a good foreground/background pixel classification by applying deep learning techniques can be divided in those that focus on successful background modeling and those that use this technique in the classification part. An extensive review can be found in [15].

#### 2.3.1. Background Modeling

Successful techniques for background modeling using deep learning include those based on auto-encoder networks [16]; networks based on fully convolutional networks used for semantic segmentation, such us U-Net; and generative adversarial networks [17].

As an example of usinh these techniques. we can mention the work of Sultana et al. [17]. They made an unsupervised visual-feature-learning hybrid GAN followed by a semantic inpainting network, which is divided into three different parts. The first one is centered on obtaining motion information for all frames by employing dense optical flow. Using it, fast moving foreground objects are identified and eliminated from the estimated background, leaving behind some missing regions. In the second step, a context encoder is trained on scene-specific data. This is used for estimating context of missing regions and is based on two constraints: global context (hybrid GAN model trained on scene-specific data) and local texture, for texture optimization (VGG-19 network). Finally, a threshold on the difference between estimated background and current frame is considered for obtaining the segmentation. This algorithm achieves a good performance in most cases, but still fails when the background has complex structures or foreground objects are very large. All these background modeling techniques rely on learning the background model for a specific scene, and thus cannot be easily updated to adapt to continuous changes in the background, incorporation of moving objects that remain static, etc. For this reason, although they provide excellent results with available databases, they are hard to apply to real scenarios conditions.

#### 2.3.2. Background Subtraction

In trying to improve traditional methods, many researchers have applied deep learning for solving the problem of background subtraction, too. Most of these works are based on traditional CNNs, such as Cascaded CNN [18] and the ConvNet trained on scene-specific data that Braham and Droogenbroeck presented [26]. In this case, the authors used as background model a single image, and then trained the network to produce a foreground probability for each pixel using as input to the network’s two patches centered at the pixel corresponding to the background image and the current image. Lim and Keles [27] used an encoder–decoder CNN trained with a few labeled frames of the scene. Another example is the method of Babaee et al. [19] which uses a background image for performing background subtraction from the incoming frames. This is done by considering matching pairs of image patches from the background image and the video frames and feeding them to a CNN that reassembles the patches into a complete output frame. While the first one is trained on the test sequence, the second one trains with a subset of all the sequences of the database, which are used both for training and testing.

More recently, the segmentation problem has also been tackled by using generative adversarial networks. By considering the work of Phillip et al. [23] as a basis, *BScGAN* [20] was presented as a method for performing background subtraction and obtaining a foreground mask for each frame. In this method, the background model is a background image which is computed as the median of a set of images from the sequence which are considered as background. For the classification network, the structure of generator and discriminator from [23] is mostly preserved: the first part is based on a U-Net [28] architecture, and the second one is formed by four convolutional and downsampling layers. In this algorithm, both original image and background model are fed as input to the generator to obtain a foreground mask. Then, this is compared to the ground truth foreground mask by the discriminator, which decides whether it is real or fake based on a learned loss function. The obtained results have been extraordinarily good, outperforming most of the previously proposed algorithms on the datasets used. However, the method uses half of each sequence for training and tests it on the other half of the exact same sequence, meaning that the method lacks generalization and requires annotations for most frames in the sequence. Moreover, a good background image estimation is required as input. In the system presented, a unique background image is used for the whole sequence. However, many scenarios cannot be correctly modeled by a single image and require more complex, adaptive models to accommodate to the variations in the scene. A more recent work [29] combines a generative adversarial network and a variational auto-Encoder in a multi-task framework, which requires training with unlabeled images of the test domain.

Another concurrent method that tries to tackle the same problem by using GANs is the one of Zheng et al. [30].

Currently, the main challenge is the development of systems based on convolutional neural networks that are context-unuspervised, and thus do not need to be trained on the test scenes. Mandal et al. [31] proposed a network with 3D convolutions in several steps to estimate the background, perform motion saliency estimation, and finally, perform foreground segmentation. They performed tests in both scene-dependent and scene-independent contexts. Tezcan et al., in [32,33], proposed a foreground segmentation network using as input the current frame and two background images of the scene, together with their foreground probability. The foreground probability map is computed with a semantic segmentation network trained to identify 150 different classes, from which the ones that usually correspond to foreground objects are selected.

The system proposed in this paper is a new context-unsupervised approach which combines an statistical approach with a CNN, thereby taking advantage of the main features of both approaches. Furthermore, adversarial loss is introduced in the CNN to obtain more consistent foreground objects. Table 1 compares the main features of the related references.

## 3. Foreground Segmentation Network

**Figure 1:**
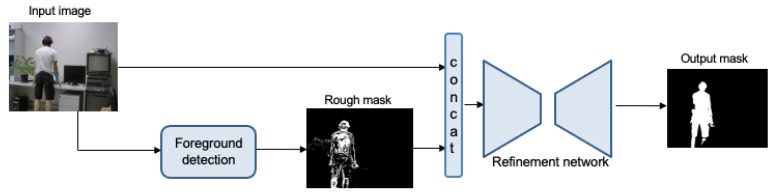
Pipeline of the mask refinement network at test time.

The foreground segmentation network proposed in this paper is composed of two sub-systems. With the objective of obtaining a generic network that does not require learning from a specific scene, it is designed as a two-step procedure. The first step detects the foreground objects according to the reasoning applied for the specific scenario, using traditional background modeling techniques and an exception to the modeling approach. This step provides rough detection which is used as input to our proposed refining network that is only trained once with a public domain labeled database, and that can be applied to any scenario without further adjustment. This network adapts an image-to-image translation network to act as a refinement network, by conditioning the result on the rough mask. Let us note that the data augmentation step proposed is also crucial to obtain this generalization of results with the reduced databases available. The global system thereby combines the segmentation criteria and adaptability to background changes of the method used for the rough approximation with the characteristic power and capability of deep learning techniques for producing a much more precise segmentation. The pipeline of the system is shown in Figure 1.

### 3.1. Foreground Detection

For rough foreground object detection, a general method with a very low computational load has to be used. Although it is perfectly plausible to substitute this detection module for any other more sophisticated system, by using a common and widely available system, the focus is centered on the improvements of the refinement network. As previously discussed, the foreground detection is usually based on the combination of a per-pixel statistical background model estimation and a per-pixel exception to the model for foreground detection. This exception to the model is usually approached as a Bayesian classification. Different methods have been considered, all of them using background models that are updated over time, but which allow the introduction of specific application criteria in this first stage. For instance, shadow suppression, or a bias to higher recall, or higher precision. These methods also contain user-defined parameters which allow for a faster adaptation to background changes, and the introduction in the background model of foreground objects that remain static or the degree of variability of the background, depending on the scenario. For the construction of a general framework, a method based on [5] has been selected, but with an adaptation of the update equations, and which automatically selects the number of Gaussian components for each pixel model [11]. In [21], the results are also compared with other two foreground detection methods.

### 3.2. Refinement Network

Inspired by the works of semi-supervised video object segmentation [34], our refinement network produces the foreground object segmentation guided by an input mask. In [34], a convnet designed for semantic image segmentation was used to produce instance segmentation in a video object segmentation context. For each new video frame, the network is guided towards the object of interest by feeding in the previous frame mask estimate. In this case, the segmentation of the object in the first frame is manually provided. The network is trained with annotated images in order to produce a refined mask output for the current frame, given a rough mask estimate from the previous frame, t-1. In order to produce accurate results, the network is fine-tuned on the manual annotation provided for the first frame and its augmentations [35].

In our work the refinement network takes as input the rough mask provided by the foreground detection methods described in Section 3.1, which is concatenated to the current frame. A convolutional neural network designed for semantic segmentation is trained to refine this rough mask, given the current input image. Unlike [34], no training is performed on the specific sequence; thus, no manual annotation is required at test time. Furthermore, given the large difference between training and testing data, we propose to use an adversarial network to achieve better generalization properties, by introducing the regularization effect of a discriminative network. For that, the image-to-image translation network of [23] is adapted to work as a refinement network. For this adaptation, the input to the network uses not only the image to segment, but also the rough mask, which affects the two parts of the network (generator and discriminator), as will be explained in the following. In this way, the image-to-image translation is conditioned on the rough mask. While in [23] the authors show very good results for graphics tasks, the usage of an adversarial loss did not improve their results for vision tasks such as semantic segmentation. We will show that in our binary segmentation task the adversarial loss is useful when conditioning on the rough mask.

**Figure 2:**
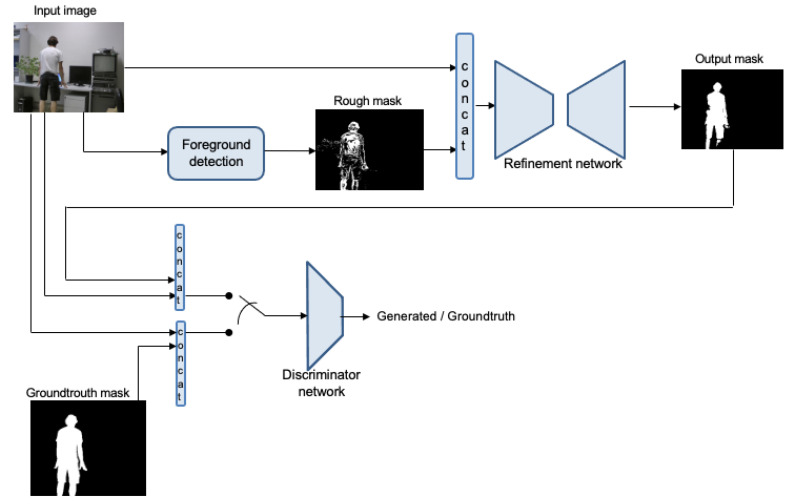
Pipeline of the complete scheme for training the refinement network.

**Figure 3:**
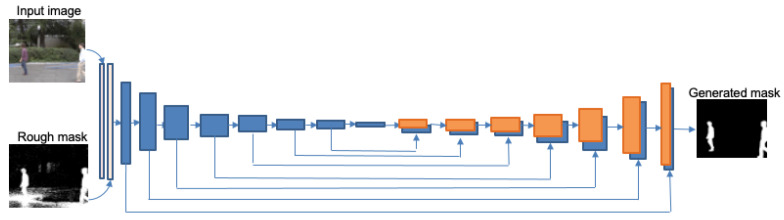
Scheme of the generator.

The network is composed of two separate parts: the generator G, which uses a semantic segmentation network scheme, and the discriminator D. In our case the first one is a guided semantic segmentation network which is trained to classify pixels in two classes (foreground and background), and the second one tries to identify the input as the ground truth or the output of the segmentation network. A complete scheme is represented in Figure 2. As in *pix2pix* [23] and *BScGAN* [20], the structure of the generator is that of a U-Net. It is divided in an encoder and a decoder with eight layers each, and a skip-connection is considered between all layers *i* and 16−i. However, in our case, the encoder is fed by two concatenated images, resized to 256 × 256, corresponding to the current RGB image to segment, and the rough binary mask approximation obtained by the foreground detection block. The output is a single image of the same size, corresponding to the refined mask. The encoder is made of eight convolutional layers with 64, 128, 256, 512, 512, 512, and 512 filters. All of them have leaky ReLu as the activation function, and batch normalization is applied to all but the first layer. For the decoder, the same structure with a reversed number of filters is applied, except changing leaky ReLu activation functions for ReLu ones, and it is followed by a last convolution, for producing the output image, and a *sigmoid* function. For its first three layers, during the training, a dropout of rate 50% is added to the previously mentioned configuration, for adding a certain degree of stochasticity avoiding overfitting. The convolutions in all layers are 4 × 4 spatial filters applied with a stride of two. The scheme of this network is represented in Figure 3.

**Figure 4:**
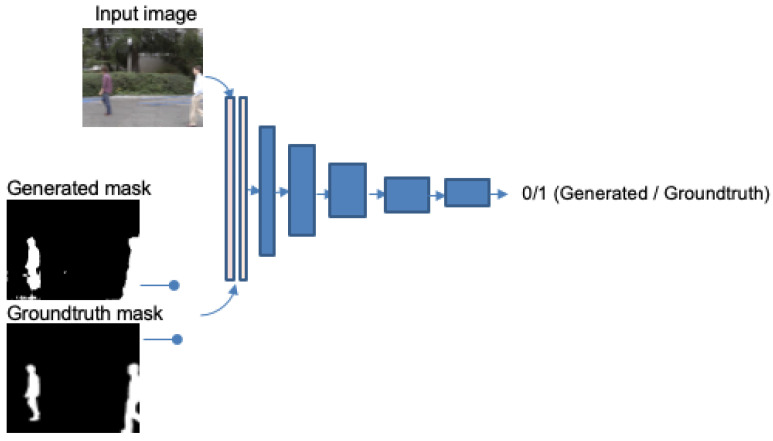
Scheme of the discriminator.

Different options for the input of the discriminator network are proposed in [22]. We use a concatenation of foreground mask (which alternates generated and ground truth) and the input image. The discriminator network is much simpler than the generator one, and it works with patches of size 70 × 70. It has five convolutional layers, followed by a sigmoid one that outputs 0 when the image is detected as generated or 1 when identified as ground truth. The number of filters in the first layer is 64 and it is multiplied by two in every layer, except for the last one, which uses just one filter for mapping to a 1-dimensional output to enter the sigmoid function. As in the generator, batch normalization is used in all except for the first layer, all convolutions are made with 4 × 4 filters and using a stride of 2, and leaky ReLu is considered for every layer, with slope of 0.2. A simplified scheme is represented in Figure 4.

The objective function of the network has two components. On the one hand, there is the output loss of the segmentation network, with input image *x*, target *y*, and output image $G\left(x\right)=\hat{y}$. With *y* being a binary mask, we chose the binary cross entropy (BCE) loss (Equation (1)) at this stage, differently from the L1 or L2 distances used in [20,23]. This loss is combined with the classical objective of an adversarial network (Equation (2)) [23,24].

(1) $$L_{BCE}\left(G\right)=I\;E_{x}\left[-\mathrm{yln}\hat{y}+\left(1-y\right)\ln\left(1-\hat{y}\right)\right]$$

(2) $$O_{AN}\left(G,D\right)=I\;E_{x,y}\left[\log\left(D\left(x,y\right)\right)\right]+I\;E_{x}\left[\log\left(1-D\left(x,G\left(x\right)\right)\right)\right]$$

When minimizing the adversarial network objective, the generator learns how to create masks that fool the discriminator, and when maximizing it, the discriminator is taught to better distinguish between ground truth and network generated masks. Then, the best generator is obtained as the one that satisfies Equation (3) [23], λ being a constant parameter.

(3) $$G^{*}=\arg\min_{G}\max_{D}\left(O_{AN}\left(G,D\right)+\lambda L_{BCE}\left(G\right)\right)$$

The training of the whole network alternates, backpropagating through one or the other subnetwork in each step. As in [24], G is trained to maximize log(D(x, G(x)) instead of minimizing log(1−D(x, G(x))). When optimizing the discriminator, the real ground truth image is first given to it as input and the weights are backpropagated. Then a fake generated image is fed to the discriminator, and backpropagation is performed again. All input images’ values are normalized between 1 and −1 for a faster training. Data augmentation is also applied to the input images by the refinement network, which is detailed in the following.

### 3.3. Data Augmentation

As will be described in the next section, databases available for training are large in their numbers of frames, but small in their numbers of videos. As a consequence, there is a high risk of overfitting to the training scenarios. To reduce this effect, a large data augmentation method is introduced, using the Albumentations package [36]. To each frame the following operations are applied: horizontal flipping (50% probability), scaling (up to 0.5), rotation (20 degrees limit), shifting, random cropping to 256 × 256, Gaussian noise addition, perspective transformation, blurring, brightness, and contrast transformation.

## 4. Results

### 4.1. Datasets

Public video datasets for background subtraction are scarce, due to the high cost of labeling videos. Reference [37] presents a comprehensive account of these datasets. From those, only three are large-scale and cover a wide range of challenging scenarios. Two of these datasets were used for our experiments: CDNet [38,39] and LASIESTA [40]. The first one was used for training the network and finding the optimal set of parameters, and the second, which is smaller, was used for testing and comparing with other state-of-the-art methods. Table 2 reports the main features of these datasets. The third large dataset, not used in this work, is mainly constituted of synthetic videos.

CDNet contains a total of 53 video sequences, divided into 11 categories, representing challenging scenarios, such as dynamic background, hard shadows, low frame-rate or night videos, among others. Each of these categories contains from 4 to 6 sequences of 600 to 7999 frames with spatial resolutions varying from 320 × 240 to 720 × 576. Annotation for some frames is given for each sequence, and the ground truth of the other frames is just used internally on its webpage for evaluating the presented methods. For this reason, all parameters were tuned using a partition of these frames with public annotations. For the sake of diversity, this partition was made by randomly picking one sequence of each category for validation and the others for training, with the result being 11 sequences in the first subset and 42 in the latter, maximizing the amount of frames used for training but maintaining the diversity in the validation subset. Once again, for each of these sequences, just the frames with provided annotations were considered. Actually, for faster training and to make use of the redundancy that is always present in videos, just one of every two frames from the annotated training sequences were considered.

In order to prove the applicability of the network in new datasets without further training or adaptation, a different dataset was used to perform further tests. LASIESTA was used for that. This dataset contains 10 different categories, also corresponding to challenging scenarios, such as camouflage, occlusions, illumination changes, rainy conditions, snowy conditions, and sunny ones. From these categories, six are focused on indoor scenarios, and the other four are outdoor ones. For each of these categories, two sequences with lengths ranging between 225 and 1400 frames are given. These sequences have a spatial resolution of 352 × 288 and contain a maximum of three moving objects each, these being either people or vehicles. For each frame in the LASIESTA database, an annotation image is given. In it, each of the foreground moving objects is annotated. When one becomes static, it has a different label assigned, and only the moving objects while they are moving are considered for the evaluation of the foreground detection methods in [40].

For assessing the performance of background subtraction algorithms, the standard evaluation metrics are used: precision, recall, and F-measure. While precision indicates what proportion of pixels detected as foreground are correct, recall gives a measure for the quantity of actual foreground pixels that are correctly classified by the method, and F-measure gives a good general perspective of both criteria, thereby being the one used for ranking methods and deciding the best one to use.

### 4.2. Experiments

The system was implemented in Python, using OpenCV and Pytorch libraries, In the first place, the train–validation partition of CDNet described was used in all hyper-parameter search experiments for the refinement network. The network thus obtained can be used in a general scenario. Results are compared and the one providing the best results is used for the remaining tests. Results for our CDNet validation partition and for LASIESTA database are provided.

#### 4.2.1. Hyper-Parameter Search

The hyper-parameter search aimed to find the best join learning rate for the generator and the discriminator and the lambda value in Equation 3. After the experiments, the Adam solver, with a learning rate of 0.0001, and a batchsize of 5, was used for both the segmentation network and the discriminator. A lambda value of 0.001 provided the best results for the validation partition, showing that the BCE loss of the generator is the most important one, and the adversarial loss improves the consistency of the results. Observation of the loss curves shows convergence to a low loss value for the generator and a high value for the discriminator after 20 epochs.

#### 4.2.2. Effect of Adversarial Network

**Figure 5:**
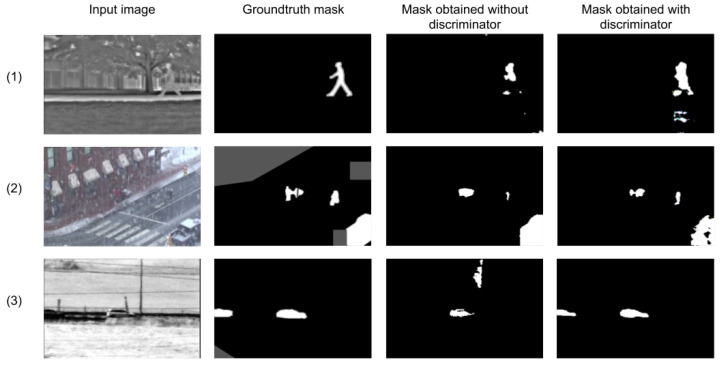
Examples of the masks generated by the network with and without the usage of a discriminator. First row: frame from the sequence “Park” (“thermal” category). Second row: frame from “wetSnow” (“badWeather” category). Third row: frame from “turbulence3” (“turbulence” category). First column: the input image of the frame is shown. Second column: its corresponding ground truth. Finally, the third column is for results obtained without a discriminator, and the last column shows results obtained using a discriminator.

The results of the refinement network without the discriminator are compared. That is, only the generator was used, a semantic segmentation network for the foreground and background classes, based on the minimization of the BCE at its output. While no significant changes can be observed in the F-score achieved for the training and validation sets, two benefits were obtained:

- Higher F-scores were achieved faster with the discriminant network, implying a better generalization of the network.
- The discriminator BCE loss introduces a comparison to the annotation that is not just pixel-wise, but takes into account the whole image for improving the result. This results in a network that has more compact foreground detections (with less or smaller holes). Examples with especially challenging situations are shown in Figure 5. More examples can be seen in the results of next section.

#### 4.2.3. Comparison to State-of-the-Art Methods

In the first place, we compare the results for CDNet dataset. The best combination of initial algorithm for the rough segmentation, set of hyper-parameters, and network scheme provided, for the CDNet dataset, a 0.93 F-score for the training partition and 0.82 for the validation partition. As mentioned, this validation partition was created by randomly selecting one sequence from each of the 11 categories. The F-score of 0.82 can be compared to the result of 0.52 obtained by the classic technique [11], which was used for the rough mask computation. This result cannot be directly compared to those obtained by other authors that used a context-independent CNN, since all these systems used for training a part of this database (for being the larger one), but each author performed different partitioning. However, a qualitative comparison can be made. The authors of [31] obtained a 0.86 F-score, those of [32], 0.78, and those of [33], 0.85.

Then, the performance of this network was assessed on the test dataset (LASIESTA). This last set was made of 20 different video sequences corresponding to 10 different challenging scenarios. Some of these scenarios, such as rainy and snowy conditions, were similar to the ones in the training partition, for which the network was expected to generalize and obtain good performance values. Other categories, such as occlusions and camouflage, did not have an equivalent category in the training or validation partition, so slightly lower performance could be expected in those sequences. For testing these hypothesis, the evaluation metrics in each of the different categories are provided. These results are compared with the ones obtained by the input masks for the network, and with those of state-of-the-art methods.

**Figure 6:**
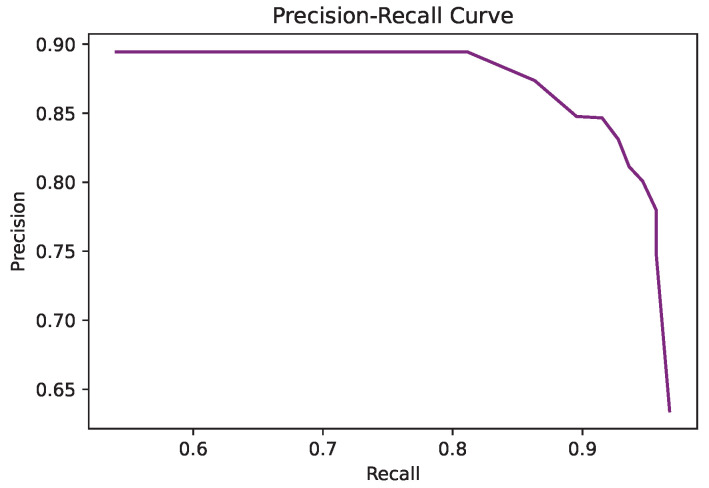
Precision vs. recall for the LASIESTA database.

First of all, Figure 6 presents the precision vs. recall curve obtained for the whole dataset when considering different thresholds for the sigmoid function between 0.01 and 0.99. It can be observed that the detection is robust to variations of this threshold, that the values of precision and recall are well balanced, and the best F-measures were obtained for thresholds between 0.4 and 0.6. The performance for each category is presented in Table 3 for a threshold of 0.5.

The F-measure obtained for each of the categories and the average for all sequences are reported in Table 4. In this table, the performances for all the methods that have been reportedly tested on this dataset are given, making use of the results published in [40]. They were all run with a single set of parameters for all the sequences and without any kind of training on this same dataset.

The evaluation method used was the one proposed on the database’s official webpage, in which only proper background pixels and foreground pixels corresponding to moving objects are considered. Additionally, the metrics for the masks that were used as input to the mask refinement network are also provided. They have been obtained with the algorithm from [11] described in Section 2.1. These initial masks were computed by initializing the background on the first half of the sequence for the masks of the second half, and initialized on the second halves of frames for the results in the first half of the sequence. The last row of the table corresponds to the output of the method developed in this work.

In the following, the methods compared are briefly described. Wren’s method [41] is one of the most popular moving object detection strategies, commonly known as the running Gaussian average. It models each pixel with a Gaussian PDF; it has a low computational cost and few parameters. While it works correctly with simple sequences and even with noisy conditions, such as “Rainy” or “Cloudy” sequences, it fails in more difficult situations, such as “Snowy” and “Bootstrap.” A similar conclusion can be derived from the results of Staufer [5], which models each pixel with a Gaussian mixture model with the aim of representing different dynamic backgrounds. It is one of the most used traditional background subtraction methods. Zivkovik [11] is an improved version of the previous reference, as described in Section 2.1. However, although it improves results in some scenarios, such as “Simple sequences,” “Occlusions,” and “Bootstrap,” it is worse for "Snowy conditions" or "Illumination changes," thereby only improving 3% on average. Maddalena [42] built an adaptive model for the background using a neural network. Although it is an adaptive method which is robust to moving backgrounds and gradual illumination changes, it obtains poor results with the “Illumination changes” and “Bootstrap” sequences. However, it provides the best results for some categories, such as the “Simple sequences,” “Occlusions,” and "Cloudy conditions." Haines [43] is based on the use of Dirichlet process Gaussian mixture models to estimate the background pixels’ model, and is one of most recent non-learning based methods to provide good results. As can be seen in the table, it provides very good results except for the “Snowy conditions” and “Bootstrap sequences.” Cuevas [14] is a non parametric kernel density estimation method that improves the results of parametric techniques such as [5] or [41] in scenarios where pixel variations cannot be described parametrically. The systems in [31,33] were recently and are based on CNNs, which, as the system proposed here, have been trained on a part of CDNet and tested on LASIESTA. The first one uses 3D convolutions, and the second one makes use of a semantic segmentation network, selecting those objects that usually correspond to foreground. Furthermore, it introduces spatio-temporal data augmentations to enrich the training partition in videos.

By comparing our results with these state-of-the-art methods, we can claim that the presented mask refinement network introduces a valuable approach. While it obtained a more than 82% F-measure in all categories, it was the one with the best performance in “Camouflage”, “Bootstrap”, and “Snowy Conditions” categories, showing quite an improvement, especially for the “Bootstrap” category. The impressive improvement in the “Bootstrap” category with respect to the previous methods is due to the introduction of a deep learning network that can obtain equally good performance for all frames in a sequence. Other methods in the ranking and also the one used for initializing the mask refinement network need a certain number of frames without any foreground object for a proper background modeling. In this case, however, the mask refinement is able to take the results generated from a bad background initialization and refine them in order to overcome the difficulties the previous methods had. This behavior is not only reflected in those few categories, but also in the overall performance of the method. The masks it obtains reach an F-measure of 87%, outperforming the previous state-of-the-art methods on this dataset.

As shown, the main advantage of the proposed method over the state-of-the-art is its ability to generalize. While the other methods provide very good results with specific kinds of sequences, they always fail in some category. For instance, [42], although providing the best results for some categories, only achieved 21% (F-measure) for the “Illumination changes” category.

Let us also mention that, while all these techniques are able to run in real-time by adjusting spatial and time resolution, the proposed scheme is particularly efficient in terms of computation. In test conditions, the inference time would include the execution of the initial foreground detection and the refinement network. The initial foreground detection used in the experiments corresponds to [10], implemented through OPENCV (MOG2), which is one of the fastest algorithms according to [44]. With a spatial resolution of 352 × 288, it achieves around 190 fps on a standard desktop computer. The processing time of the refinement network depends on the availability of GPUs. On standard GPU, it is easy to achieve 75 fps [45]. Thus, the refinement network drastically increases the performance of the initial segmentation, without compromising a real-time execution.

**Figure 7:**
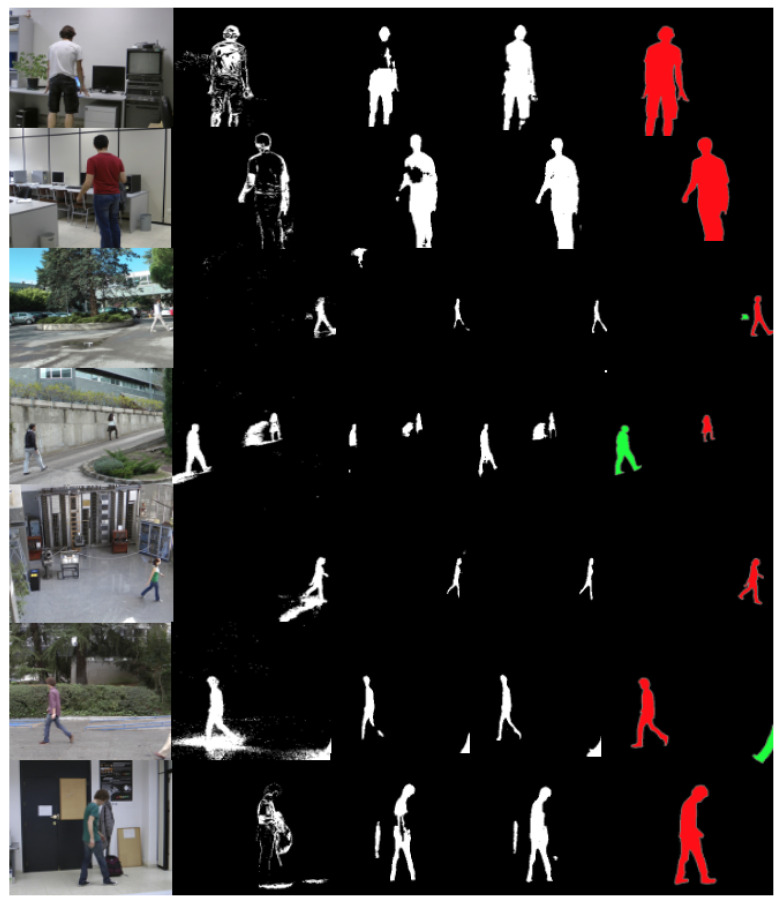
Examples for the evaluation of the mask through the mask refinement method with LASIESTA database. First column shows original images. Second column, input rough mask provided by [11]. Third row, results of the network using the semantic segmentation network without adversarial loss, as in [21]. Forth column shows the results of this paper. Last column shows the ground truth masks.

Qualitative results can be seen in Figure 7, which presents some examples to analyze the improvement obtained with the networks designed. The second column of this figure shows the input masks provided by [10], which are used as rough input to the network, as shown in Figure 1, and third column shows the output of the network trained without the adversarial loss, as in [21]. The initial mask in second row suffers mainly from the camouflage effect and shadows, whereas the results of the refinement network without the adversarial loss present some holes in the masks in areas not detected by the initial mask. However, the complete network, with the adversarial loss, produced more compact results, shown in forth column. By analyzing these results, it can be observed that the network learned how to properly generalize to unknown sequences even of a different nature than the ones used for training. The network has actually learned how to refine the masks obtained with a classical method, reducing its noise, filling up holes in figures, and discarding the background pixels that were mistakenly detected as foreground.

## 5. Discussion and Future Work

Two main aspects of the background subtraction approach that has been proposed can be further analyzed: the rough mask computation method and the effects of the training database.

Regarding the rough mask computation, in our previous work we compared three standard approaches, and the one providing the best results was used for the continuation of the work, focused on the improvement of the refinement network in a general framework. However, it is important to note that minor improvements of the rough mask are directly translated into the final results. Furthermore, the statistical methods for computation of the rough mask can be adapted to different scenarios. For instance, shadow suppression methods are more important in outdoor scenarios. Lower precision at the cost of higher recall can be accepted in some surveillance systems. For this reason, in order to make the most of the proposed technique, the appropriate rough mask computation method should be chosen.

Concerning the training database for the refinement network, we believe that better results could be obtained if specific scenes were discarded. The training database CDNet has very different categories, which obtain different results in the validation. Some of them are hard scenarios which might have a bad influence on the overall results. Future work could be to analyze the effect of the inclusion of some of these sequences in the training dataset. Furthermore, given the limited size of training datasets for video, the new data augmentation technique for videos recently proposed in [33], which has boosted the results obtained by [32], could be included to improve the results.

## 6. Conclusions

This paper presented a novel approach for successfully subtracting the background from any sequence without requiring the annotation for any of its frames, or specific scene training. Most previous methods attempting this task use a huge amount of annotations and specific training for each individual sequence. Our method uses a semantic segmentation network adapted to classify into two classes, foreground and background. It is composed of two steps: The first one uses a conventional foreground detection method to provide a rough approximation to the masks. Then, the CNN uses this approximation as a guidance and outputs a refined foreground mask.

Different classical methods for obtaining the rough mask that constitutes the input of the network can be used. Using the best method and the optimal set of parameters, the network was trained on one dataset (CDNet) and tested on another one (LASIESTA), obtaining satisfactory results. One of the advantages of the proposed method is that the method for the computation of the rough mask can be easily substituted by a more specific or advanced one, thereby boosting the final results.

It was also shown that the use of an adversarial network can improve the results of the semantic segmentation network in this task by improving the generalization properties of the obtained network, capturing more detail in the foreground masks, and detecting more compact foreground components. Therefore, it was shown that this training acts as a higher-level loss function that takes into account global properties of the obtained images, in contrast to the local ones obtained by conventional loss functions.

## Figures and Tables

**Table 1:** Analysis of foreground segmentation methods.

	Background Modeling	Background Subtraction	Statistical	CNN	GAN	Context Dependent
2,3,4,7,8	X		X			
5,6,9,10,11,12,13,14	X	X	X			
16	X			X		X
17	X			X	X	X
18,19,26,27		X	X	X		X
20,28,29,30		X		X	X	X
31	X	X		X		
32,33		X		X		
Ours	X	X	X	X	X	

**Table 2:** Main features of CDNet and LASIESTA datasets.

	CDNet	LASIESTA
Sequences	53	20
Categories	11	10
Indoor sequences	8	14
Outdoor sequences	45	10
Frames/sequence	600–7999	225–1400
Resolution	320 × 240–720 × 576	352 × 288
Labeled images	1 out of 10	All

**Table 3:** Performance in precision, recall, and F-measure for the LASIESTA dataset. Categories are: simple sequences (SI), camouflage (CA), occlusions (OC), illumination changes (IL), modified background (MB), bootstrap (BS), cloudy conditions (CL), rainy conditions (RA), snowy conditions (SN), and sunny conditions (SU). Last column shows the Average values.

	SI	CA	OC	IL	MB	BS	CL	RA	SN	SU	AV
Prec	0.77	0.86	0.74	0.76	0.94	0.85	0.84	0.85	0.89	0.93	**0.83**
Rec	0.94	0.93	0.94	0.9	0.92	0.87	0.85	0.95	0.89	0.82	**0.92**
F-meas	0.84	0.89	0.83	0.82	0.93	0.86	0.84	0.90	0.89	0.87	**0.87**

**Table 4:** Reported F-measures for the best methods published on LASIESTA’s official website. The last two rows correspond to the performances of the input to the mask refinement network (input mask in Figure 2) and the generated masks (output mask in the same Figure) with the network proposed. The results are given as final averages for all the sequences in the database (last column), and independently for each of the categories in the dataset.

	SI	CA	OC	IL	MB	BS	CL	RA	SN	SU	AV
Wren [41]	0.82	0.76	0.89	0.49	0.74	0.47	0.86	0.85	0.60	0.75	**0.72**
Stauffer [5]	0.83	0.83	0.89	0.29	0.76	0.36	0.87	0.78	0.60	0.72	**0.69**
Zivkovik [11]	0.90	0.83	0.95	0.24	0.87	0.53	0.88	0.88	0.38	0.71	**0.72**
Madd. [42]	**0.95**	0.86	**0.95**	0.21	0.91	0.40	**0.97**	0.90	0.81	**0.88**	**0.78**
Haines [43]	0.89	**0.89**	0.92	0.85	0.84	0.68	0.83	0.89	0.17	0.85	**0.78**
Cuevas [14]	0.88	0.84	0.79	0.65	**0.94**	0.66	0.93	0.87	0.78	0.72	**0.80**
Mandal [31]	0.86	0.49	0.93	0.85	0.79	0.87	0.87	0.87	0.49	0.83	**0.79**
Tezcan [33]	0.92	0.68	0.96	**0.88**	0.81	0.77	0.93	**0.94**	0.84	0.79	**0.85**
MOG [10]	0.70	0.68	0.72	0.41	0.65	0.55	0.57	0.67	0.38	0.71	**0.60**
Ours	0.84	**0.89**	0.83	0.82	0.93	**0.86**	0.84	0.90	**0.89**	0.87	**0.87**

## Data Availability

Not applicable.
